# Evaluation of psychological and physiological predictors of fatigue in patients with COPD

**DOI:** 10.1186/1471-2466-9-47

**Published:** 2009-10-21

**Authors:** Agnieszka Lewko, Penelope L Bidgood, Rachel Garrod

**Affiliations:** 1 Faculty of Health and Social Care Sciences, St. George's University of London, London, UK; 2 Kingston University, Cranmer Terrace, SW17 0RE London, UK; 3 Faculty of Computing, Information Systems and Mathematics, Kingston University, Penrhyn Road, KT1 2EE Kingston upon Thames, Surrey, UK; 4 Kings College NHS Foundation Trust, Dulwich Hospital, SE22 8PT London, UK

## Abstract

**Background:**

Fatigue in COPD impairs functional status; however there are few studies examining mechanistic pathways of this symptom. The aims of this study are to compare fatigue between COPD patients and healthy age-matched subjects, and to identify predictors of fatigue in COPD.

**Methods:**

Seventy four COPD patients, mean age 69.9 (49-87) yrs, mean (SD) % predicted FEV_1 _46.5 (20.0) % and FEV_1_/FVC ratio 0.45 (0.13) and 35 healthy subjects, mean age 67.1 (50-84) yrs completed the Multidimensional Fatigue Inventory (MFI 20). Patients' assessment included Depression (HADS), lung function, BMI, muscle strength, incremental shuttle walk test (ISWT), exercise oxygen saturation (SpO_2_), Borg breathlessness (CR-10) and exertion (RPE). Serum level of Interleukin 6 (IL-6) was recorded. Differences in MFI 20 between groups were examined and predictors of fatigue identified using logistic regression.

**Results:**

Significant differences (p < 0.01) were found between the COPD and healthy subjects for all MFI 20 dimensions. There were significant differences when classified according to GOLD and dyspnoea stages for selected dimensions only. Predictors of General Fatigue were depression, muscle strength and end SpO_2 _(R^2 ^= .62); of Physical Fatigue: depression, % predicted FEV_1_, ISWT and age (R^2 ^= .57); Reduced Activity: % predicted FEV_1_, BMI and depression (R^2 ^= .36); Reduced Motivation: RPE, depression and end SpO_2 _(R^2 ^= .37) and Mental Fatigue: depression and end SpO_2 _(R^2 ^= .38).

**Conclusion:**

All dimensions of fatigue were higher in COPD than healthy aged subjects. Predictive factors differ according to the dimension of fatigue under investigation. COPD-RF is a multi component symptom requiring further consideration.

## Background

Fatigue, as a symptom in chronic obstructive pulmonary disease (COPD) may have diverse manifestations, such as physical or mental tiredness, loss of attention, concentration or motivation [1,2]. It is an important [3-5] and highly prevalent symptom [6,7] with data from one study suggesting that 90% of COPD patients may report fatigue [6]. Although fatigue in COPD is acknowledged by clinicians, it is often neglected. In fact, compared with cancer the predictors and patho-mechanisms of fatigue are poorly evaluated in COPD and there is a lack of understanding regarding the management of fatigue. There are evident associations between fatigue, impaired quality of life and increased depression [8-10]. Furthermore, fatigue and dyspnoea in COPD, whilst related, appear to be separate entities [11,4]. Using the FACIT-fatigue uni-dimensional scale, no association between disease severity and fatigue is noted [8]. However, the Multidimensional Fatigue Inventory (MFI 20) [12], which is well validated in COPD and shows moderate relationships with airflow obstruction [10,13]. The MFI 20 provides a multi-dimensional evaluation of fatigue, enabling investigation of the different components. Since several factors are likely to be involved in the development of COPD related fatigue (COPD-RF), the use of a multi-dimensional tool enables further elucidation of mechanistic pathways. For instance, whilst hypoxia and hypoxaemia, impaired fat free mass and loss of fatigue resistant muscle fibres are associated with fatigue [14], it is not known if the variables relate to physical or mental fatigue specifically. In healthy subjects, acute exercise fatigue is associated with raised levels of Interleukin 6 (IL6) [15,16]; possibly, this pathway is important in the development of COPD-RF [17]. There are few studies that comprehensively investigate predictors of the different domains of fatigue in COPD. This study attempts to draw together the psychological and physiological aspects of fatigue to develop working models of COPD-RF, by using comparative data from healthy age-matched subjects. This research hypothesises that increases in each component of fatigue in COPD may be predicted by differing factors. The aims of this study were firstly, to provide comparative data of fatigue, using the MFI 20, between COPD patients and healthy elderly subjects and secondly, to explore possible predictors of fatigue components of the MFI 20.

## Methods

Full ethical consent was obtained for this cross sectional study from the Royal Marsden Local Research Ethics Committee (London, UK). Patients and control subjects gave written informed consent prior to entering the study.

### Subject recruitment

**Figure 1:**
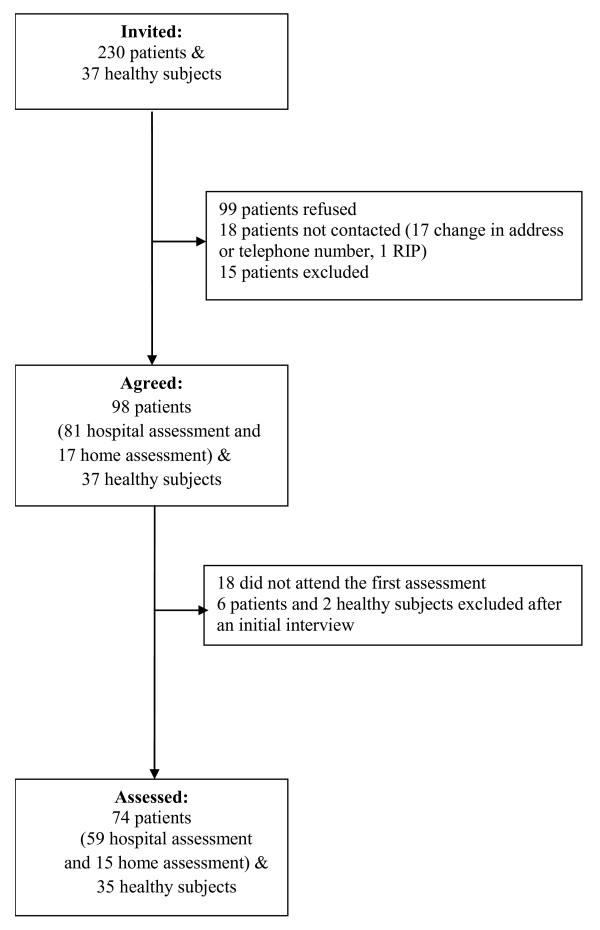
**Flow chart of recruitment and assessment**.

A total of 80 COPD patients (defined as FEV_1_/FVC ratio < 0.7 with a general practitioner or chest physician diagnosis) and 37 healthy elderly subjects were recruited to the study. Patients with stable COPD (no exacerbation or change of medication in last 6 weeks) were recruited from the chest clinic of St. George's Hospital. Figure 1 shows the patient recruitment flow chart. Healthy subjects were recruited from the staff and volunteers of St. George's University, St. George's Hospital and from an Open University group. Absence of airway obstruction was confirmed by spirometry. Additionally, patients and subjects were excluded if they had a history of significant inflammatory co-morbidities such as carcinoma, rheumatoid arthritis or stroke, unstable angina, a diagnosis of psychiatric disorders, or mobility limiting conditions.

### Assessments

Fifty nine patients included in the study attended the hospital for a baseline morning assessment of approximately 2.5 - 3.5 hours (transport available). Assessment was carried out over two days if necessary, for instance, when the patient was too tired to continue. A further 15 housebound severe COPD patients were unable to attend the hospital for assessment; for these patients an adapted home assessment was provided.

### Variables measured

#### Subjective fatigue

Fatigue was assessed in the morning prior to other assessments in patients and healthy subjects using MFI 20, a 20-item self-report validated instrument [12,18]. This tool has been previously used in COPD population and it is recommended as an outcome measure in clinical settings [19]. The questionnaire asks subjects to describe how they have been feeling lately. For issues of clarity this was specified as "within the last two weeks". The tool consists of five dimensions covering General Fatigue (GF), Physical Fatigue (PF), Reduced Activity (RA), Reduced Motivation (RM) and Mental Fatigue (MF). Each dimension has 4 items, each item scored from 1-5 with higher scores representing greater levels of fatigue.

#### Depression and breathlessness scores

All participants completed the Hospital Anxiety and Depression Scale (HADS). The HADS is a questionnaire designed to measure depressive moods and anxiety separately [20]. A score of 11 or higher indicates probable presence of the mood disorder, but it is not a synonymous of the diagnosis.

Baseline breathlessness level was assessed using the Medical Research Council Dyspnoea Grade (MRC) [21].

Health Related QoL was measured using Saint George's Respiratory Questionnaire (SGRQ) [22].

#### Physiological measures

For all participants height (cm) and weight (kg) were measured. Body Mass (BMI) and Fat Free Mass (FFMI) indices were determined from body composition measurements using a non-invasive bioelectrical impedance technique (Body composition analyser, Tanita Ltd BC-418MA, UK). Baseline FEV_1 _and FVC were assessed using a spirometer Micro Plus MS03 (Micro Medical Ltd, Rochester UK) and according to BTS recommendations [23]. GOLD classification was used to determine patients' airway obstruction severity [24].

#### Muscle strength

For all participants quadriceps maximal torque was measured using a "Cybex Norm™ Testing and Rehabilitation System". The protocol consisted of 1 minute of consecutive concentric knee extensions and flexions, performed maximally at an angular velocity of 60°/sec, following a 10-repetition trial. The break between the trial and actual test was 20 seconds. Maximum torque was recorded as the best attempt during 1 minute isokinetic work of the dominant side (FtLbs) and as FtLbs/subject bodyweight (lb) *100.

#### Exercise tolerance

For patients only, maximal exercise tolerance was assessed using the Incremental Shuttle Walking Test (ISWT), an incremental, externally paced exercise capacity test conducted according to standardised procedure [25]. Endurance exercise tolerance was then assessed using the Endurance Shuttle Walking Test (ESWT) [26]. Prior to and after each test percutaneous arterial oxygen saturation (SpO_2_) and heart rate were measured using a pulse oxymeter (Pulsox-3i-Konika Minolta, Singapore) applied to the finger. Borg CR 10 (Dyspnoea assessment) and Borg Rating of Perceived Exertion (RPE) scales were scored after each test [27,28].

#### Blood sampling

For patients only, a fasting, resting venous blood sample was obtained from the median cubital vein and Interleukin-6 (IL-6) was measured from serum. Blood samples were centrifuged within 2 hours of collection and serum was stored at -20°C until assay. The analysis was performed on an Immulite^® ^1000 automated analyser (Siemens Medical Solutions Diagnostics, formally Euro/DPC Ltd Gwynedd Wales, UK). The limit of detection for serum IL-6 was 0.2 pg/mL^-1^. Patients' haemoglobin (Hb) levels were determined from their ear lobe capillary blood sample, using blood gas/electrolyte analyser (model 5700; Instrumentation Laboratory, Lexington MA). Anaemia was defined according to WHO criteria [29].

#### Home assessment

The 15 home assessments were as described above with the exception of measures of exercise tolerance, muscle strength and blood sampling.

### Statistical analysis

Summary statistics of the healthy subjects' and COPD patients' characteristics were reported. Independent sample t-tests or Mann-Whitney tests as appropriate were used to test for differences between the patients and the healthy subjects. The Kruskal-Wallis test was used to determine differences between fatigue dimensions according to the MRC score and GOLD classification with the significance level set at α = 0.05. When significant differences were found, post hoc analysis was carried out using Mann-Whitney tests with Bonferroni correction. The data used to measure fatigue are not metric and therefore multiple linear regression techniques are inappropriate. Due to the small number of subjects, logistic rather than ordinal regression was chosen to identify predictors of the 5 dimensions of fatigue; a backward elimination method was used. Therefore for each dimension of fatigue, a binominal category was defined using a cut-off point of the highest value of fatigue in the healthy subjects. Values above this point were considered as fatigued and values below as not fatigued. Explanatory variables that were independent of one another, according to appropriate correlation tests were included in the initial regression models. Other, clinically relevant variables, as identified from literature [9,10,13,14,17,30,31] were also included. The final models, presented here, are those where all the remaining independent variables are statistically significant predictors of the fatigue dimension under consideration and which have the highest R^2 ^value. The data from 15 home assessments were excluded due to the modified assessment. All analyses were performed using SPSS 15.

## Results

### Comparative data: Fatigue in COPD subjects versus healthy older subjects

Seventy four mild-to-severe COPD patients and 35 healthy subjects completed all assessments.

There were no significant differences in fatigue according to gender in either the COPD (52 male vs. 22 female) or the healthy group (12 male vs. 23 female). Median scores for COPD patients were significantly higher than those of the healthy subjects in all dimensions of MFI 20. Table 1 shows the scores for fatigue and other variables and differences between COPD patients and the healthy subjects.

**Table 1:** Characteristics and comparison of COPD and healthy subject groups

	**COPD (n = 74)**	**Healthy (n = 35)**	**p value**
MFI 20 General Fatigue	13 (5)	8 (4)	p < 0.001

MFI 20 Physical Fatigue	16 (5.25)	7 (5)	p < 0.001

MFI 20 Reduced Activity	13.5 (6)	6 (5)	p < 0.001

MFI 20 Reduced Motivation	10 (6)	6 (4)	p < 0.001

MFI 20 Mental Fatigue	8 (7)	6 (4)	p = 0.008

% FEV_1_	46.5(20.0)	96.5(13.2)	p < 0.001

FEV_1_/FVC ratio	0.44 (0.13)	0.78(0.1)	p < 0.001

Age (yrs)	69.9 (8.4)	67.11 (8.8)	^NS^

BMI (kg/m^2^)	26.2 (5.4)	25.2 (3.4)	^NS^

FFMI (kg/m^2^)	18.2 (2.6)	17.9 (2.2)	^NS^

Depression (HADS)	6 (5)	1 (3)	p < 0.001

Anxiety (HADS)	7 (6)	3 (4)	p < 0.001

Peak Tq (% BW)	38.6 (12.0)*	51.6 (14.5)	p < 0.001

ISWT (m)	343.3 (183.9) *	-	-

Post walk SpO_2 _(%)	90.0(5.9)*	-	-

Borg exertion (RPE)	13(2.0)*	-	-

IL 6 (pg/mL) (n = 57)	5.4(5.9) *	-	-

MRC dyspnoea score	3 (2)	-	-

SGRQ	56.0 (27.4)	-	-

Data presented as median (IQR) for MFI 20-Multidimensional Fatigue Inventory, HADS - Hospital Anxiety and Depression scale, SGRQ - Saint George's Respiratory Questionnaire, total score, Borg exertion, MRC score or mean (SD) for % FEV_1 _- % predicted forced expiratory volume in one second, FEV_1_/FVC - Forced Expiratory Volume in one second/Forced Vital Capacity, BMI - Body Mass Index; FFMI - Fat Free Mass Index; Peak Tq (% BW) - quadriceps peak torque (% Body Weight), ISWT - Incremental Shuttle walk test, SpO_2 _- Percutaneous arterial oxygen saturation. *not measured in home-assessed patients (n = 59).

### Fatigue and disease severity

**Figure 2:**
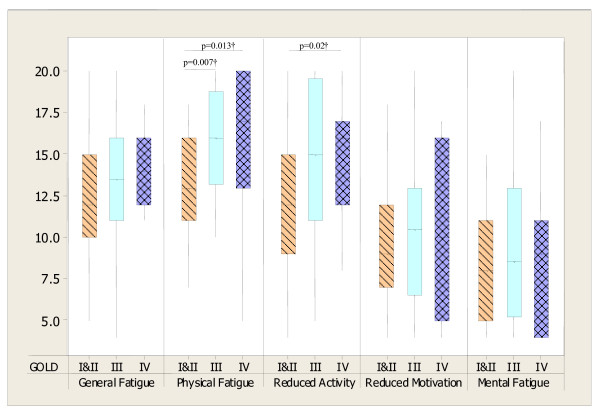
**Box plots showing MFI 20 dimensions (median and IQR) in COPD patients for GOLD stages**. GOLD stage I & II (n = 27), III (n = 32) and IV (n = 15). Kruskal Wallis tests: p = 0.008 for Physical Fatigue and p = 0.045 for Reduced Activity; p > 0.05 for General Fatigue, Reduced Motivation and Mental Fatigue; †Mann-Whitney test (Bonferroni correction p = 0.02).

**Figure 3:**
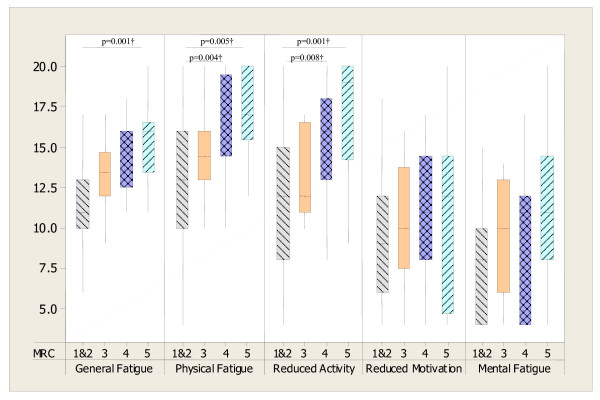
**Box plots showing MFI 20 dimensions (median and IQR) in COPD patients for MRC scores**. MRC score 1&2 (n = 31), 3 (n = 12), 4 (n = 17), 5 (n = 14). Kruskal Wallis tests: p = 0.001 for General Fatigue, p = 0.004 for Physical Fatigue, p = 0.001 for Reduced Activity, p > .05 for Reduced Motivation and Mental Fatigue; †Mann-Whitney test (Bonferroni correction p = 0.008).

Statistically significant differences in fatigue when categorised according to the GOLD and MRC dyspnoea classifications were evident for selected dimensions of MFI 20 only (see figure 2 and figure 3).

### Meaningful variables for fatigue dimensions

Regression analysis was performed on full data available from 59 patients (40 male) aged 68.7 (8.1), mean (SD) % predicted FEV_1 _48.2 (20.9) and FEV_1_/FVC ratio 0.45 (0.13). Clinically relevant variables were identified based on literature and results of correlations with fatigue dimensions. Following the initial regression analyses nine possible predictors were identified: % predicted FEV_1_, depression, muscle torque, ISWT, end exercise SpO_2_, IL6, Borg RPE, BMI, age. The final decision on which variables to include was determined by clinical relevance and by the best possible model of predictive variables.

#### Variables excluded from the analysis

The mean (SD) haemoglobin level was 15.6 (2.9) g/dL in male and 14.6 (1.6) g/dL in female patients. Only 2 patients had values below normal. Anaemia was therefore not a feature of the patient population and thus was not included in the regression analysis. There were high correlations between anxiety and depression, BMI and FFMI, ISWT and ESWT. Hence anxiety, FFMI and ESWT were not entered into the regression as independent variables as the others were considered more important clinically.

MRC score as a measure of dyspnoea was initially considered as a predictor of fatigue. Although expected from literature and from the results presented in figure 3, it was not a significant predictor for any of fatigue dimension and it was eventually excluded in the final regression process. Measures of quality of life (SGRQ) were collected to enable baseline characterisation of the patients. Although quality of life has been shown to be associated with fatigue in previous studies [8,9], direction of causality is unknown. It was felt that fatigue was more likely to be a mediator of impaired quality of life than the other direction, thus SGRQ data were not included in the regression as independent predictors.

#### Predictors of fatigue in COPD

For GF the following 5 variables were entered: depression, muscle torque, % predicted FEV_1_, end SpO_2_, IL 6; for PF: depression, ISWT, % predicted FEV_1 _and age; for RA: depression, ISWT, % predicted FEV_1 _and BMI; for RM: depression, end Borg RPE and end SpO_2 _and for MF: depression, IL6 and end SpO_2_. Table 2, 3, 4, 5 and 6 give the results of the logistic regression analyses. Table 2 shows, for instance, that the significant predictors of GF (as measured by Wald statistic) are depression, muscle torque and end exercise SpO_2_. For every increase of 1 in the depression score a patient is approximately 1.5 times more likely to be fatigued and for lower end exercise SpO_2 _there is a slightly higher chance of being fatigued; similarly lower muscle strength is associated with higher risk of fatigue.

**Table 2:** Logistic regression results for General Fatigue

**MFI 20**	**Variables included**	**Wald χ^2^**	**B (SE)**	**Odd ratio** **Exp b**	**95% CI lower-upper**
**General Fatigue** **(n = 48)**	HADS depression	8.1	0.43 (0.15)	1.54	1.14-2.07
	
	Tq %BW	5.0	-0.13 (0.06)	0.88	0.79-0.98
	
	End SpO_2_	2.9	-0.014 (0.08)	0.87	0.74-1.02
	
	Constant	3.4	13.9 (7.6)	1109057	

R^2 ^= .62 (Nagelkerke); HADS depression-depression score of hospital anxiety and depression scale, Tq%BM - quadriceps muscle torque % of body mass, End SpO_2 _- percutaneous arterial saturation post walking test

**Table 3:** Logistic regression results for Physical Fatigue

**MFI 20**	**Variables included**	**Wald χ^2^**	**B (SE)**	**Odd ratio** **Exp b**	**95% CI lower-upper**
**Physical Fatigue** **(n = 57)**	HADS depression	11.3	0.47 (0.14)	1.61	1.22-2.12
	
	%pred. FEV_1_	9.9	-0.11 (0.04)	0.90	0.83-0.96
	
	ISWT	5.2	0.01 (0.003)	1.01	1.0-1.01
	
	Age	2.8	0.9 (0.06)	1.1	0.98-1.22
	
	Constant	2.6	-7.16 (4.48)	0.001	

R^2 ^= .57 (Nagelkerke). HADS depression-depression score of hospital anxiety and depression scale, %pred. FEV_1 _- % predicted forced expiratory volume in one second, ISWT-Incremental Shuttle Walk Test

**Table 4:** Logistic regression results for Reduced Activity

**MFI 20**	**Variables included**	**Wald χ^2^**	**B (SE)**	**Odd ratio** **Exp b**	**95% CI lower-upper**
**Reduced Activity** **(n = 57)**	%pred. FEV_1_	7.5	-0.06 (0.02)	0.95	0.91-0.98
	
	BMI	4.5	0.19(0.08)	1.21	1.03-1.41
	
	HADS depression	3.9	0.19 (0.94)	1.20	1.0-1.45
	
	Constant	2.0	-3.12 (1.95)	0.04	

R^2 ^= .36 (Nagelkerke). %pred. FEV_1 _- % predicted forced expiratory volume in one second, BMI - Body Mass Index, HADS depression-depression score of hospital anxiety and depression scale

**Table 5:** Logistic regression results for Reduced Motivation

**MFI 20**	**Variables included**	**Wald χ^2^**	**B (SE)**	**Odd ratio** **Exp b**	**95% CI lower-upper**
**Reduced Motivation** **(n = 56)**	Borg exertion	5.8	0.54 (0.22)	1.71	1.11-2.66
	
	HADS depression	5.7	0.24 (0.10)	1.28	1.04-1.56
	
	End SpO_2_	4.9	-0.13(0.06)	0.88	0.78-0.99
	
	Constant	0.3	2.69 (5.28)	14.67	

R^2 ^= .37 (Nagelkerke). Borg exertion (score) post walk test, HADS depression-depression score of hospital anxiety and depression scale, End SpO_2 _- percutaneous arterial saturation post walking test.

**Table 6:** Logistic regression results for Mental Fatigue

**MFI 20**	**Variables included**	**Wald χ^2^**	**B (SE)**	**Odd ratio** **Exp b**	**95% CI lower-upper**
**Mental Fatigue** **(n = 52)**	HADS depression	7.3	0.4 (0.15)	1.49	1.12-2.0
	
	End SpO_2_	3.1	0.2 (0.12)	1.23	0.98-1.54
	
	Constant	4.3	-23.06(11.16)	0.0	

R^2 ^= .38 (Nagelkerke). HADS depression-depression score of hospital anxiety and depression scale, End SpO_2 _- percutaneous arterial saturation post walking test.

### Summary

Fatigue score was significantly higher in COPD compare to control group for all dimensions of MFI 20. After stratification for MRC and GOLD classifications there were significant differences only for selected dimensions of fatigue. Fatigue, when considered as a multi-component construct was explained by a different combination of variables.

## Discussion

The results from this study show that subjective fatigue measured with MFI 20 was significantly higher in the COPD group than in healthy elderly people. Previous work supports these findings [8,32], which suggests that the increased fatigue reflects disease entity and may therefore be considered as COPD-RF and it should be properly addressed in clinical practice. In contrast to other authors [8] differences were found in fatigue according to GOLD staging, however these differences were specific for selected dimensions of fatigue only. The difference in findings likely reflects the different fatigue tools used, with this study using a multi-dimensional tool compared with the FACIT-fatigue, a uni-dimensional tool.

In line with this, it is demonstrated here that differing fatigue dimensions can be explained by different physiological and psychological variables associated with COPD and only depression was a common predictor. Although according to MRC dyspnoea classification there were significant differences for Reduced Activities, General and Physical Fatigue, the regression analysis excluded dyspnoea score as a possible predictive variable of fatigue. Importantly, the cohort used in regression study differ from one used in MRC classification analysis and data from home-assessed most severe patients may have been more insightful. Previous studies also showed associations between dyspnoea and fatigue [8,30,31]. Nonetheless, fatigue and dyspnoea are both subjective symptoms of COPD and some of the patho-mechanisms of fatigue may be common for those of dyspnoea. Therefore, this may explain the close association between these symptoms. Since fatigue is not routinely assessed in current clinical practice, the models from this study may help to identify patients who are at risk of being fatigued and develop effective fatigue management strategies. Furthermore, the regression analyses revealed that each component could be explained by different variations. Hence, the multi-rather than uni-dimensional assessment should be considered. Over half of the variation in general fatigue was explained by a combination of depression, exercise de-saturation and muscle strength. These data then provide further support for the role of muscle training, depression management and use of supplementary oxygen in COPD. In this present study exercise de-saturation was also a significant predictor for the MFI 20 Reduced Motivation component. For Reduced Motivation; depression, post exercise saturation and exertion explained a little less than 40% of the variation. This component includes items such as 'I dread having to do things' and 'I don't feel like doing anything'. Low oxygen levels may be associated with changes in cognitive function [33,34] and stimulate affective areas of the brain, mainly in the frontal lobe, which is associated with motivational process [35,36]. Feasibly, the administration of oxygen during exertion may be associated with lower fatigue levels and enhanced motivation. To date this application of oxygen has not been explored.

For physical fatigue the best model, a combination of depression, lung function and exercise tolerance, explained 57% of the variation. This provides some explanation as to how therapies that improve exercise tolerance and depression can have an impact on fatigue in COPD [37]. Muscle strength and exercise tolerance were significant predictors for general and physical fatigue, respectively. These two variables were highly correlated and therefore only one was used in the regression analysis. Muscle weakness and reduced exercise tolerance are well recognised in the COPD population [38,39] and may be important factors influencing COPD RF.

Around 40% of the variation in Reduced Activity was explained by the combination of lung function, BMI and depression. In this instance a higher BMI was associated with greater fatigue. This probably reflects the U-shaped curve nature of the association between BMI and outcomes, both low and high BMI is generally associated with poorer outcome [40]. Nutritional status remains an important therapeutic outcome in the management of COPD. Furthermore, in physically related dimensions of fatigue the severity of airway obstruction explained a significant amount of the variance, suggesting that treatments which affect airflow obstruction may also benefit the perception of physical fatigue. For mental fatigue few COPD patients were identified as fatigued and the results for this component should be treated with caution.

Anaemia may be one of the causes of fatigue and can be present in COPD patients [41]; however, haemoglobin levels were generally within the normal range in the cohort, thus this factor could not be included in this analysis. Nevertheless, it is clear from this study, that COPD RF remains a significant problem even in patients with moderate disease and no anaemia. Although some variables were not included in the final regression model, they may still play a role in the development of COPD RF. For example, there was a strong correlation between anxiety and depression, but it was decided only to include depression. Similarly, ESWT was strongly related to ISWT but only the maximal test was included. Although IL6 was considered a possible predictor, none of the final regression models included it, which may be due to its correlation with both muscle strength and walking distance [42] or reflects the need for other measures such as receptors.

Previous studies using a multiple regression identified depression as a predictive variable of uni-dimansional fatigue in COPD [8,30]. However, findings here reveal that depression is a predictor for all dimensions of fatigue.

## Conclusion

This study shows that all dimensions of fatigue are greater in COPD than in healthy people of a similar age range. Increased fatigue in this population is therefore a feature of COPD and not of age per se. COPD-RF is a multi component construct and as such different aspects of fatigue are influenced by different clinical manifestations. Comprehensive treatment of COPD-RF includes management of depression, muscle weakness, optimisation of BMI and exercise de-saturation levels. In contrast to other authors it was found that fatigue differs according to GOLD staging, however this is relevant only for Physical Fatigue and Reduced Activity. This study goes some way towards explaining the mechanistic pathway of COPD-RF and provides information to target hitherto neglected treatments.

## Abbreviations

BTS: British Thoracic Society; COPD: Chronic Obstructive Pulmonary Disease; COPD-RF: Chronic Obstructive Pulmonary Disease Related Fatigue; CR 10: Borg Dyspnoea Assessment Scale; ESWT: Endurance Shuttle Walking Test; FEV_1_: Forced Expiratory Volume in One Second; FtLb: Foot-Pound Force; FVC: Forced Vital Capacity; GOLD: Global Initiative for Chronic Obstructive Lung Disease; HADS: Hospital Anxiety and Depression Scale; Hb: Haemoglobin; IL 6: Interleukin 6; ISWT: Incremental Shuttle Walking Test; MRC: Medical Research Council Dyspnoea Grade; MFI 20: Multidimensional Fatigue Inventory; GF: General Fatigue Subscale; MF: Mental Fatigue Subscale; PF: Physical Fatigue Subscale; RA: Reduced Activity Subscale; RM: Reduced Motivation Subscale; RPE: Borg Rating of Perceived Exertion Scale; QoL: Quality of Life; SGRQ: Saint George's Respiratory Questionnaire; SpO_2_: Percutaneous Arterial Oxygen Saturation; Tq: Torque; % BW: Percentage of Body Weight.

## Competing interests

This project was part of AL's PhD, which was in part funded by GlaxoSmithKline (GSK). RG has received honorariums and conference support from Pfizer, GSK, Hill-Rom and Boehringer Ingelheim (not specifically related to this study), PLB has no competing interests.

## Authors' contributions

AL has contribution to conception and design, data collection and interpretation; PLB provided substantial contribution to interpretation of data, manuscript revision and RG participated in the design and manuscript writing. All authors read and approved the final manuscript.

## Pre-publication history

The pre-publication history for this paper can be accessed here:



## References

[B1] Armes J, Krishnasamy M, Higginson IJ (2004). Fatigue in cancer.

[B2] Del Fabbro E, Dalal S, Bruera E (2006). Symptom control in palliative care - Part II: Cachexia/anorexia and fatigue. Journal of Palliative Medicine.

[B3] Brissot R, Gonzalez-Bermejo J, Lassalle A, Desrues B, Doutrellot PL (2006). Fatigue and respiratory disorders. Annales de Readaptation et de Medecine Physique.

[B4] Small S, Lamb M (1999). Fatigue in chronic illness: the experience of individuals with chronic obstructive pulmonary disease and with asthma. Journal of Advanced Nursing.

[B5] Yeh ML, Chen HH, Liao YC, Liao WY (2004). Testing the functional status model in patients with chronic obstructive pulmonary disease. Journal of Advanced Nursing.

[B6] Kinsman RA, Yaroush RA, Fernandez E, Dirks JF, Schocket M, Fukuhara J (1983). Symptoms and experiences in chronic bronchitis and emphysema. Chest.

[B7] Walke LM, Byers AL, Tinetti ME, Dubin JA, McCorkle R, Fried TR (2007). Range and severity of symptoms over time among older adults with chronic obstructive pulmonary disease and heart failure. Archives of Internal Medicine.

[B8] Baghai-Ravary R, Quint JK, Goldring JJP, Hurst JR, Donaldson GC, Wedzicha JA (2008). Determinants and impact of fatigue in patients with chronic obstructive pulmonary disease. Respiratory Medicine.

[B9] Breslin E, Schans C Van der, Breukink SO, Meek P, Mercer K, Volz W, Louie S (1998). Perception of fatigue and quality of life in patients with COPD. Chest.

[B10] Breukink SO, Strijbos JH, Koorn M, Koeter GH, Breslin EH, Schans CP Van der (1998). Relationship between subjective fatigue and physiological variables in patients with chronic obstructive pulmonary disease. Respiratory Medicine.

[B11] Gift AG, Shepard CE (1999). Fatigue and other symptoms in patients with chronic obstructive pulmonary disease: do women and men differ?. Journal of Obstetric, Gynecologic, & Neonatal Nursing.

[B12] Smets EM, Garssen B, Bonke B, De Haes JC (1995). The Multidimensional Fatigue Inventory (MFI) psychometric qualities of an instrument to assess fatigue. Journal of Psychosomatic Research.

[B13] Oh EG, Kim CJ, Lee WH, Kim SS (2004). Correlates of fatigue in Koreans with chronic lung disease. Heart & Lung.

[B14] Evans WJ, Lambert CP (2007). Physiological basis of fatigue. American Journal of Physical Medicine & Rehabilitation.

[B15] Robson-Ansley PJ, De Milander L, Collins M, Noakes TD (2004). Acute interleukin-6 administration impairs athletic performance in healthy, trained male runners. Canadian J Appl Physiol.

[B16] Spath-Schwalbe E, Hansen K, Schmidt F, Schrezenmeier H, Marshall L, Burger K, Fehm HL, Born J (1998). Acute effects of recombinant human interleukin-6 on endocrine and central nervous sleep functions in healthy men. Journal of Clinical Endocrinology & Metabolism.

[B17] Gan WQ, Man SF, Senthilselvan A, Sin DD, Gan WQ, Man SFP, Senthilselvan A, Sin DD (2004). Association between chronic obstructive pulmonary disease and systemic inflammation: a systematic review and a meta-analysis. Thorax.

[B18] Smets EM, Garssen B, Cull A, De Haes JC (1996). Application of the multidimensional fatigue inventory (MFI-20) in cancer patients receiving radiotherapy. British Journal of Cancer.

[B19] Meek PM, Lareau SC (2003). Critical outcomes in pulmonary rehabilitation: assessment and evaluation of dyspnea and fatigue. Journal of Rehabilitation Research & Development.

[B20] Zigmond AS, Snaith RP (1983). The hospital anxiety and depression scale. Acta Psychiatrica Scandinavica.

[B21] Fletcher CM (1960). Standardised questionnaire on respiratory symptoms: a statement prepared and approved by the MRC committee on the aetiology of chronic bronchitis; MRC breathlessness score. BMJ.

[B22] Jones PW, Quirk FH, Baveystock CM, Littlejohns P (1992). A self-complete measure of health status for chronic airflow limitation. The St. George's Respiratory Questionnaire. American Review of Respiratory Disease.

[B23] British Thoracic Society, BTS (1994). Guidelines for the measurement of respiratory function. Respiratory Medicine.

[B24] Global Initiative for Chronic Obstructive Lung Disease (GOLD) (2006). Global Strategy for the Diagnosis, Management, and Prevention of Chronic Obstructive Pulmonary Disease.

[B25] Singh SJ, Morgan MD, Scott S, Walters D, Hardman AE (1992). Development of a shuttle walking test of disability in patients with chronic airways obstruction. Thorax.

[B26] Revill SM, Morgan MD, Singh SJ, Williams J, Hardman AE (1999). The endurance shuttle walk: a new field test for the assessment of endurance capacity in chronic obstructive pulmonary disease. Thorax.

[B27] Borg GA (1982). Psychophysical bases of perceived exertion. Medicine & Science in Sports & Exercise.

[B28] Borg GA (1998). Borg's Perceived exertion and pain scales. The Borg RPE Scale.

[B29] World Health Organisation, WHO (2001). Iron Deficiency Anemia. Assessment, Prevention, and Control. Geneva, Switzerland.

[B30] Kapella MC, Larson JL, Patel MK, Covey MK, Berry JK (2006). Subjective fatigue, influencing variables, and consequences in chronic obstructive pulmonary disease. Nursing Research.

[B31] Woo K (2000). A pilot study to examine the relationships of dyspnoea, physical activity and fatigue in patients with chronic obstructive pulmonary disease. Journal of Clinical Nursing.

[B32] Theander K, Unosson M (2004). Fatigue in patients with chronic obstructive pulmonary disease. Journal of Advanced Nursing.

[B33] Grant I, Prigatano GP, Heaton RK, McSweeny AJ, Wright EC, Adams KM (1987). Progressive neuropsychologic impairment and hypoxemia. Relationship in chronic obstructive pulmonary disease. Archives of General Psychiatry.

[B34] Kozora E, Filley CM, Julian LJ, Cullum CM (1999). Cognitive functioning in patients with chronic obstructive pulmonary disease and mild hypoxemia compared with patients with mild Alzheimer disease and normal controls. Neuropsychiatry, Neuropsychology, & Behavioral Neurology.

[B35] Incalzi RA, Marra C, Giordano A, Calcagni ML, Cappa A, Basso S, Pagliari G, Fuso L (2003). Cognitive impairment in chronic obstructive pulmonary disease: A neuropsychological and spect study. Journal of Neurology.

[B36] Sari A, Oshiata S, Toriumi T, Yamashita S, Kojima S, Kakumoto S, Yonei A (1992). Cerebral blood flow and cerebral oxygen consumption in patients with COPD on mechanical ventilation. Intensive Care Medicine.

[B37] Lacasse Y, Martin S, Lasserson TJ, Goldstein RS (2007). Meta-analysis of respiratory rehabilitation in chronic obstructive pulmonary disease. A Cochrane systematic review. Europa Medicophysica.

[B38] Celli BR, MacNee W, The ERS Task Force (2004). Standards for the diagnosis and treatment of patients with COPD: a summary of the ATS/ERS position paper. European Respiratory Journal.

[B39] Gosselink R, Troosters T, Decramer M (2000). Distribution of muscle weakness in patients with stable chronic obstructive pulmonary disease. Journal of Cardiopulmonary Rehabilitation.

[B40] Landbo C, Prescott E, Lange P, Vestbo J, Almdal TP (1999). Prognostic value of nutritional status in chronic obstructive pulmonary disease. Journal of Respiratory and Critical Care Medicine.

[B41] Shorr AF, Doyle J, Stern L, Dolgitser M, Zilberberg MD (2008). Anemia in chronic obstructive pulmonary disease: epidemiology and economic implications. Current Medical Research & Opinion.

[B42] Garrod R, Marshall J, Barley E, Fredericks S, Hagan G (2007). The relationship between inflammatory markers and disability in chronic obstructive pulmonary disease (COPD). Primary Care Respiratory Journal.

